# Visual Comparative Omics of Fungi for Plant Biomass Deconstruction

**DOI:** 10.3389/fmicb.2016.01335

**Published:** 2016-08-24

**Authors:** Shingo Miyauchi, David Navarro, Igor V. Grigoriev, Anna Lipzen, Robert Riley, Didier Chevret, Sacha Grisel, Jean-Guy Berrin, Bernard Henrissat, Marie-Noëlle Rosso

**Affiliations:** ^1^Aix-Marseille Université, INRA, UMR1163, Biodiversité et Biotechnologie FongiquesMarseille, France; ^2^CIRM-CF, UMR1163 Biodiversité et Biotechnologie FongiquesMarseille, France; ^3^US Department of Energy Joint Genome Institute, Walnut CreekCA, USA; ^4^Plateforme d’Analyse Protéomique de Paris Sud-Ouest, UMR1319 Micalis, INRAJouy-en-Josas, France; ^5^Architecture et Fonction des Macromolécules Biologiques, CNRS, Aix-Marseille UniversitéMarseille, France; ^6^INRA, USC 1408 AFMBMarseille, France; ^7^Department of Biological Sciences, King Abdulaziz UniversityJeddah, Saudi Arabia

**Keywords:** self-organizing maps, data visualization, multi-omics integration, LPMOs, lignocellulosic biomass, biorefinery, CAZymes

## Abstract

Wood-decay fungi contain the cellular mechanisms to decompose such plant cell wall components as cellulose, hemicellulose, and lignin. A multi-omics approach to the comparative analysis of wood-decay fungi gives not only new insights into their strategies for decomposing recalcitrant plant biomass, but also an understanding of how to exploit these mechanisms for biotechnological applications. We have developed an analytical workflow, Applied Biomass Conversion Design for Efficient Fungal Green Technology (ABCDEFGT), to simplify the analysis and interpretation of transcriptomic and secretomic data. ABCDEFGT utilizes self-organizing maps for grouping genes with similar transcription patterns, and an overlay with secreted proteins. The key feature of ABCDEFGT is simple graphic outputs of genome-wide transcriptomic and secretomic topographies, which enables visual inspection without *a priori* of the omics data and facilitates discoveries of co-regulated genes and proteins. Genome-wide omics landscapes were built with the newly sequenced fungal species *Pycnoporus coccineus*, *Pycnoporus sanguineus*, and *Pycnoporus cinnabarinus* grown on various carbon sources. Integration of the post-genomic data revealed a global overlap, confirming the pertinence of the genome-wide approach. ABCDEFGT was evaluated by comparison with the latest clustering method for ease of output interpretation, and ABCDEFGT gave a better biological representation of fungal behaviors. The genome-wide multi-omics strategy allowed us to determine the potential synergy of particular enzymes decomposing cellulose, hemicellulose, and lignin such as Lytic Polysaccharide Monooxygenases, modular enzymes associated with a cellulose binding module1, and Class II Peroxidase isoforms co-regulated with oxido-reductases. Overall, ABCDEFGT was capable of visualizing genome-wide transcriptional and secretomic profiles for intuitive interpretations and is suitable for exploration of newly-sequenced organisms.

## Introduction

The wealth of genomic and transcriptomic information from filamentous fungi has revealed an unexpected striking diversity of lignocellulosic enzymes encoded in their genomes (Floudas et al., 2015). The regulation of genes coding for cellulases and hemicellulases in response to isolated plant polysaccharides has been extensively studied in a few model fungi (Amore et al., 2013). However, further studies are necessary in order to understand the fine-tuned enzymatic deconstruction of complex natural biomasses made of diverse chemical composition.

Saprotrophic white-rot fungi are wood decayers able to break down major plant cell wall polymers such as cellulose, hemicelluloses, and lignin to retrieve the carbon necessary to their growth (Blanchette, 1991; Riley et al., 2014). Such abilities are related to the secretion of a wide range of enzymes that may be exploited to improve the degradation of complex plant biomass for carbon recycling and biofuel production (e.g., Couturier et al., 2012). The genus *Pycnoporus* is known for the efficient degradation of lignin, and the use of the fungi for biotechnological applications has been reviewed (Eggert et al., 1997; Gupta et al., 2011; Lomascolo et al., 2011).

Enzymes active on plant biomass polymers including glycoside hydrolases (GH), carbohydrate esterases (CE), polysaccharide lyases (PL), and auxiliary activity enzymes (AA) are classified in sequence-based families in the Carbohydrate Active Enzyme database (CAZy^1^, Levasseur et al., 2013; Lombard et al., 2014).

The volume of next-generation sequencing (NGS) data has been increasing rapidly. For example, there are several hundreds of fungal genomes publically available from MycoCosm (Grigoriev et al., 2014). These data have been used to study the portfolio of genes coding for enzymes involved in wood decay and to analyze the evolution of these gene repertoires associated with various life styles and environments (Floudas et al., 2012, 2015; Riley et al., 2014). Beyond gene repertoires, fungal transcriptomic studies may provide information on the genes actively expressed in specific conditions, enabling better understandings of the molecular mechanisms of wood decay (Hori et al., 2014).

Next-generation sequencing is particularly useful to capture a quantitative genome-wide picture of the transcribed genes. However, typical statistical analysis of NGS data produces complex outputs that are often difficult to interpret from a biological point of view. Also, extracting biological knowledge from the ocean of data is a challenge. Thus, the development of customized tools is desirable for data mining. The ideal tools would be able to simplify the complexity of input data, facilitate comparisons of biological responses from various conditions, and produce user-friendly visual outputs for intuitive interpretations.

Analysis of grouped genes from multiple conditions is useful as similar expression profiles may be indicative of co-functionality of genes (Si et al., 2014). However, finding an appropriate clustering tool for RNA-seq data is not easy despite there are various NGS analytical tools available (Yang and Kim, 2015).

Weighted gene co-expression network analysis (WGCNA) is a method that constructs correlation networks of genes. However, a large number of samples per condition are required to produce reliable results for multiple comparisons (Langfelder and Horvath, 2008). This requirement is not satisfied for the typical RNA-seq setup where three biological replicates are used per condition.

K-means clustering has been applied for the microarray-based fungal gene expression analysis (MacQueen, 1967). Although it is an established statistical algorithm, it has a particular limitation for clustering. The algorithm separates given input data into k partitions where k is an input parameter that is difficult to determine when external constraints of the input data are unknown (Tan et al., 2014). Algorithms used for microarray analysis are not applicable to RNA-seq data due to the different nature of output data. For this reason, a Poisson and negative binomial modeled method MBCluster.Seq has been developed (Si et al., 2014).

Self-organizing map (SOM) is an unsupervised data-driven machine learning algorithm. This algorithm constructs a neural network with given input data. The method is used to simplify high-dimensional data because it reduces the number of features by grouping similar items and forming clusters (Kohonen, 1982). This makes it suitable for complex large data such as RNA-seq data. Conveniently, the SOM algorithm has a unique feature of making two-dimensional maps in contrast to the conventional clustering methods creating dendrograms (Lloyd, 1982; Langfelder et al., 2008; Si et al., 2014).

Since the nature of SOM is data-driven, it is possible to generate neural networks of genes and identify condition-specific responses in transcriptomes of newly sequenced organisms with limited gene annotations. The flexible graphics of SOMs can be exploited to visualize genome-wide transcriptomic data. SOM has been used in NGS based epigenetic and transcriptomic studies (Steiner et al., 2012; Kim et al., 2013). The practical applications for large-scale omics data using SOM have been reviewed (Binder and Wirth, 2015).

The comparative analysis of fungal multi-omics data gives new insights into the dynamics of the enzymes produced by the fungi during decomposition of complex plant materials (Hori et al., 2014). The selection of secreted fungal enzymes could therefore be significant for biotechnological applications. In particular, co-regulated genes coding for enzymes simultaneously produced by the fungi could inspire the design of new enzyme cocktails for efficient plant biomass deconstruction. Additionally, comparative analyses without *a priori* assumptions may lead to identification of as-yet-unknown co-regulated genes and proteins potentially involved in lignocellulose deconstruction. For these reasons, we created an integrated-omics tool to investigate the mechanisms of plant biomass conversions in newly sequenced fungi.

The workflow, Applied Biomass Conversion Design for Efficient Fungal Green Technology, (ABCDEFGT), was designed to perform omics data mining for co-regulated genes and corresponding secreted proteins which are potentially involved in the degradation of complex plant materials. Our workflow emphasizes on visualization of the data for intuitive data mining of genes/proteins of interest. The workflow was demonstrated with three newly sequenced fungal species *Pycnoporus coccineus* (Couturier et al., 2015), *Pycnoporus sanguineus* (Miyauchi et al., in preparation), and *Pycnoporus cinnabarinus* (Levasseur et al., 2014). We performed information mining of the fungal omics data from both transcriptomic and secretomic point of view. Our approach was evaluated by examining the concordance of the resultant gene/protein clusters in terms of biological relevance. Also, our method was compared to the model-based clustering (i.e., MBCluster.Seq; Si et al., 2014). To the best of our knowledge, this is the first study in which the genome-wide transcriptome and secretome of fungi were systematically clustered to generate layers of omics topographies for biological extrapolations.

## Results

**FIGURE 1:**
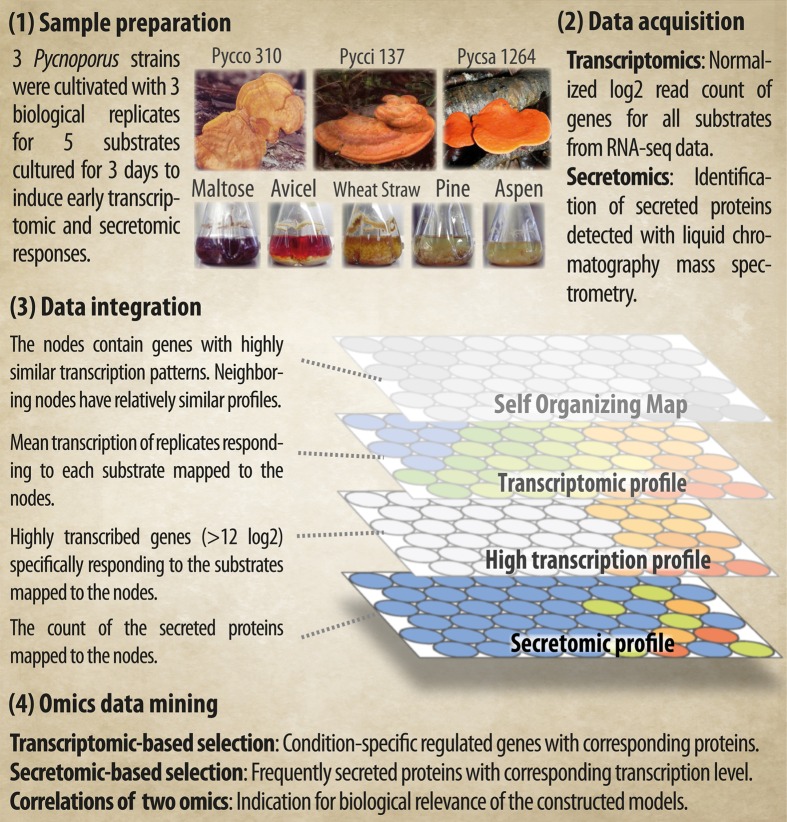
**Overview of the experimental design and the integration of the transcriptomic and secretomic data using the Applied Biomass Conversion Design for Efficient Fungal Green Technology (ABCDEFGT) workflow**.

Three fungal strains, *Pycnoporus coccineus* CIRM-BRFM 310 (Pycco 310), *Pycnoporus cinnabarinus* CIRM-BRFM 137 (Pycci 137), and *Pycnoporus sanguineus* CIRM-BRFM 1264 (Pycsa 1264) were grown on each of the five carbon sources selected. Two carbon sources were used as control conditions; maltose, which is easily assimilated by fungi and Avicel, which was used as a cellulose model substrate. Ground wheat straw, pine wood, and aspen wood were used as models for gramineae biomass, softwood biomass, and hardwood biomass, respectively (**Figure 1**).

A visualization workflow for omics integration was developed for the datasets to identify fungal genes and proteins involved in lignocellulose deconstruction. The integrated omics model consists of; (1) genes with similar transcription patterns being clustered into nodes; (2) the calculation of the node-wise mean of the normalized transcript read counts, which reflects the transcription level in response to each condition; (3) the selection of condition-specific highly transcribed gene clusters; and (4) the count of secreted proteins detected from the culture medium (**Figure 1**). All topographies are comparable as the positions of the nodes are fixed in the maps, enabling the simultaneous visual inspection of complementary biological information (**Figure 1**).

### The Integrated Omics Model of *Pycnoporus coccineus* CIRM-BRFM 310

**FIGURE 2:**
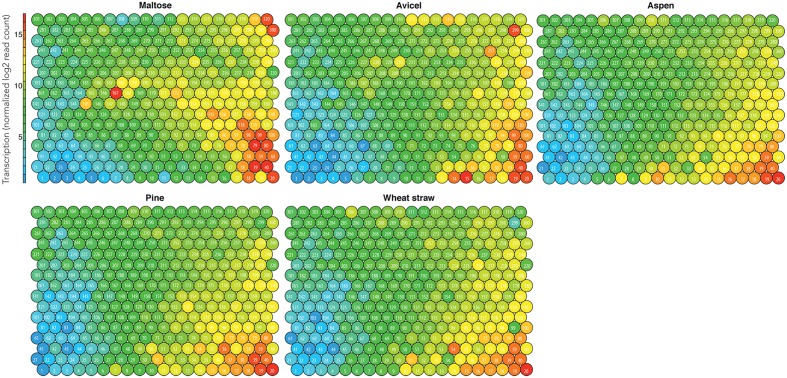
**Integrated omics model of Pycco 310.** Mean transcription of biological replicates for the individual substrates. The node identification is labeled (i.e., 1–320).

**FIGURE 3:**
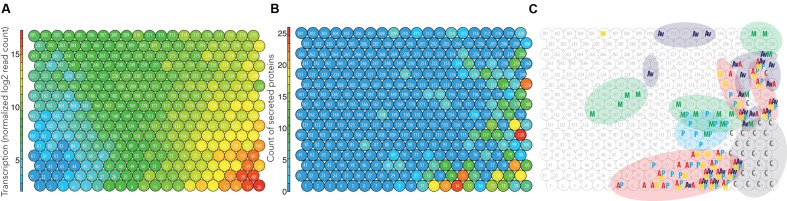
**Integrated omics model of Pycco 310.** The node identification is labeled (i.e., 1–320). **(A)** Global mean transcription levels per node combined from the five cultivation conditions. **(B)** The count of the total proteins secreted per node indicates secretion hotspots. **(C)** Groups of highly transcribed genes (>12 log2 read count) were labeled and highlighted in circle. C, Common to all substrates; M, Maltose; Av, Avicel; W, Wheat straw; P, Pine; A, Aspen.

We produced models for Pycco 310 (**Figure 2**) and two other strains (**Supplementary Figures S1** and **S2**). The mid-level transcribed nodes in orange, yellow, and light green (12–14 log2 mean transcription) included the genes up-regulated on pine, aspen, or wheat straw (**Figure 2**). Similarly regulated genes were highlighted in circles in order to visually separate the gene groups (**Figure 3C**). This approach was found to be effective because gene clusters with similar transcriptomic patterns had neighboring locations in the topography. This is due to the hexagram format of SOM, which produced six related gene clusters in proximity, and enabled us to neatly visualize the transcriptomic patterns of the genes in response to the different types of substrates.

The nodes in red (>15 log2 mean transcription) at the right bottom corner were the genes most highly transcribed (**Figure 3A**). Because these nodes were highly transcribed under all growth conditions including the control condition, maltose (**Figure 3C**), we considered these nodes as essential for housekeeping processes. Since these particular nodes were not specific for degradation of the testing substrates (i.e., aspen, pine, wheat straw), this aspect was not investigated further.

Globally, the areas of nodes with above 10 log2 read count (right side of **Figure 3A**) coincide with nodes with higher counts of secreted proteins (right side of **Figure 3B**). This trend was observed also for the other two strains Pycci 137 and Pycsa 1264 (**Supplementary Figures S1** and **S2**). The correlation coefficients of transcriptomic and secretomic profiles for the three strains were approximately 0.55 (*p* < 0.001; **Supplementary Table S1**; **Supplementary Data S1**). The moderate correlation might be explained by; (1) the nodes with around 12 log2 mean transcription mainly contain the high number of proteins (**Figures 3A,B**); (2) the most highly transcribed nodes (>15 log2 mean transcription) contain genes that are considered for housekeeping and information on such intracellular proteins is absent in the secretomic data; (3) the omics data used were fungal early responses to the substrates on the third day of cultivation. The corresponding proteins from the highly expressed genes might not have been sufficiently accumulated in the culture medium for detection at the time point.

### Selection of Genes Regulated in Substrate-Specific Manners

Our strategy was to overlay complementary information from genome-wide transcriptomics and the secretomics of corresponding gene products. Our intention was to investigate the genes regulated in response to lignocellulosic substrates (wheat straw, pine, and aspen). Such genes were potentially involved in the deconstruction of the complex linkages such as hemicellulose and lignin intra- and inter-chain linkages. For this purpose, we filtered the genes that were both specifically highly transcribed and differentially expressed on lignocellulosic substrates as compared to the two simple carbon sources, maltose, and cellulose.

In the case of Pycco 310, we identified nodes that contained genes highly transcribed on wheat straw, pine, or aspen (>12 log2) but not on maltose or cellulose. The genes that were significantly up-regulated on complex plant substrates (log2 fold change > 1; adjusted *p* < 0.05), in comparison to maltose, were further selected from the nodes (**Table 1**).

**Table 1:** Pycco 310 genes up-regulated on complex plant substrates.

Substrate	Nodes	Genes	Secreted/predicted	CAZymes	Unknown
Aspen	15	121	22/49 (45%)	23	16
Pine	21	224	24/58 (41%)	20	18
Wheat straw	16	105	16/34 (67%)	14	10

***Nodes:** The number of substrate-specific nodes containing genes highly transcribed only on the plant substrates. **Genes**: The number of genes highly transcribed and differentially regulated in the substrate-specific nodes. **Secreted/predicted**: The number of secreted proteins detected in the culture medium at least in one growth condition/the number of proteins with predicted signal peptides (as determined by SignalP). **CAZymes** (carbohydrate active enzymes): The number of predicted proteins similar to carbohydrate active enzymes. **Unknown**: The number of proteins with predicted secretion and no predicted function.*

We identified seven nodes commonly selected from the transcriptomic data generated from wheat straw, pine, and aspen culture conditions (the full list is in **Supplementary Data S2**). Of these, particularly noteworthy nodes were 12, 16, 31, and 239.

Some nodes contained genes coding for predicted CAZymes active on hemicellulose and CAZymes active on pectin. There were two CAZymes potentially active on hemicellulose (GH12; protein ID 1358049 and GH43; 1435501) and one active on pectin (GH28; 688728) in node 12 (**Supplementary Data S2**). The presence of these three CAZymes within a single node suggests that the fungus simultaneously produces these enzymes to cleave hemicellulose and pectin polymers of the plant biomass. A similar co-regulation pattern was observed in node 52 where genes were highly transcribed and up-regulated on aspen including a xylanase gene (GH10; 1435885) and pectin-active enzymes such as CE8 (1438246) and GH28 (1377553; 1446047).

Auxiliary activity 2 peroxidases are involved in lignin breakdown and often work in concert with other oxidoreductases such as glucose–methanol–choline (GMC) oxidoreductases (CAZy family AA3; Hernández-Ortega et al., 2012). Two AA2 peroxidases (1468611; 1436321) in node 31 and the AA3 (1368318) in node 16 could have been involved in lignin breakdown since they all show up-regulation on the three substrates. Cooperative activity has also been shown between AA2 peroxidases and AA5_1 glyoxal oxidases (Kersten and Cullen, 2014). One predicted AA5_1 glyoxal oxidase (1480943, node 74) had its gene regulated on wheat straw and aspen, suggesting that it could have synergistic activity with the above-mentioned AA2 peroxidases. In addition, a coordinated breakdown for lignin and hemicellulose might have occurred in our cultures with the presence of two AA2 genes (1431101; 1464049), a GH10 xylanase (1395316) and CE1 acetylxylan esterase (1377160) found together in a node (node 9).

The genes coding for non-secreted proteins were enriched in node 239; this trend was seen across the three complex substrates. Non-secreted proteins specifically up-regulated on pine were found in nodes 94, 113, 114, 133, and 197. These genes may be expressed intracellularly for metabolic adaptation of the fungus to the plant biomass. For example, the genes (protein ID : 1436381; 1439874; 1521754) encoding predicted cytochrome P450s in nodes 94, 113, and 239 may have a role in detoxifying toxic compounds resulting from the decomposition of pine (**Supplementary Data S2**).

Additionally, we identified genes coding for predicted secreted proteins with unknown functions. Some of these genes are co-regulated with CAZyme genes and could participate in the breakdown of the plant biomass such as protein ID 1363452 and 1364528 in node 12, 1438837, and 1425076 in node 16, and 1439153 in node 52. Such genes could be of interest for further functional studies.

In conclusion, the genes clustered based on similar transcription profiles. Differential transcription was observed from the growth on complex plant substrates in comparison to maltose and cellulose. The result gave enzymes potentially active in synergy on hemicellulose, pectin, and lignin polymers. Since enzymes active on plant biomass polymers are often members of multi-copy gene families, this strategy was particularly effective to identify the individual gene copies used by the fungus in our growth conditions. Finally, the integration of whole-transcriptome data enabled the identification of metabolic adaptations potentially related to plant substrate deconstruction, including detoxification mechanisms.

### Selection of Nodes Enriched in Secreted Proteins

We identified sets of co-regulated genes coding for abundantly secreted proteins that potentially act synergistically for the degradation of the substrates. We observed that the nodes containing genes highly transcribed on cellulose (Avicel) and highly transcribed on pine, aspen, or wheat straw tended to have the highest number of secreted proteins (**Figure 3B**). Also, the mean transcription of the genes in response to Avicel was mostly higher than any other substrates (**Table 2**; **Supplementary Table S2**). This observation is consistent with the study on saprotrophic fungi, in which cellulose is perceived as a signal for the transcription of a wide range of genes coding for enzymes active on cellulose and hemicellulose (Amore et al., 2013).

**Table 2:** Pycco 310 nodes most enriched in secreted proteins and their corresponding transcriptome profiles.

ID	Proteins	Mal	Avi	Asp	Pin	Whs	CAZyme
120	26	12	15	13	13	13	GH47, GH92, GH27, GH17, GH5_22, PL8_4
14	24	7	15	14	13	13	CBM1-GH6, CBM1-GH5_7, AA9-CBM1, GH7, AA8-AA3_1, GH115, AA9-CBM1, CBM1-CE16, GH131-CBM1
35	23	7	12	13	12	13	GH28, Lysophospholipase, GH3, GH45, Peptidase S10, Peptidase S53
220	23	7	13	12	10	9	GH7, CE16, GH12, CBM1-CE1, CBM1-GH3, CBM1-GH5_5, CBM1-CE15, CBM1

***ID:** Identification of the nodes from the integrated omics model **(Figure 3)**. **Proteins**: Total number of secreted proteins detected from the culture medium. **Mal/Avi/Asp/Pin/Whs**: Mean log2 read count of the genes per node in response to maltose, Avicel, aspen, pine, or wheat straw. **CAZyme**: Predicted functions of the secreted proteins based on CAZy and KOG annotations.*

Four nodes showing the highest number of proteins detected in the secretome were selected from the secretomic topography (**Figure 3B**). Interestingly, most of the genes from the selected nodes coded for CAZymes. In particular, CAZymes associated with a Carbohydrate Binding Module 1 (CBM1) were abundant in nodes 14 and 220. CBM1 is a protein domain mainly found in fungi that has affinity to crystalline cellulose and improves access of the enzyme catalytic module to the substrate (Guillén et al., 2010). The identified CBM1-containing CAZymes are active on cellulose (AA9-CBM1, CBM1-GH5_5) and glucan polymers (GH131-CBM1, CBM1-GH6, and CBM1-GH3). An enrichment of such CAZymes in the selected nodes was consistent with up-regulation of the genes in response to cellulose-derived stimuli (**Table 2**). It was observed that enzymes active on the branching linkages from the hemicellulose polymer backbone were clustered together with cellulose-active enzymes (e.g., GH115, CE16, CE1, and CE15), suggesting the fungal response to cellulose and plant biomass-derived stimuli involves a diverse set of enzymes acting in synergy on a variety of linkages.

### Comparison of the Clustering Methods

We measured the biological relevance of our model in comparison to MBCluster using the Pycco 310 data. Correlations were estimated between the average transcription levels of the individual gene clusters and the corresponding count of the secreted proteins (**Supplementary Data S3**). Given that in general a higher gene transcription level corresponds to a higher expression of gene products, we expected some positive correlation if a clustering method is biologically relevant.

Self-organizing map and MBCluster were used separately to generate 320 gene clusters. This number of clusters was optimal for SOM and gave a resolution of about 35 genes/cluster. The correlation coefficients were 0.55 and 0.24 (*p* < 0.001) for SOM and MBCluster, respectively, suggesting that SOM combined the transcriptomic and secretomic data more effectively than MBCluster.

An additional attempt was made to increase the correlation coefficient for MBCluster by arbitrarily lowering the number of clusters to 100, giving 112 genes/cluster with the correlation coefficient 0.31 (*p* < 0.001). This coefficient was still lower than that of SOM with the high resolution of 35 genes/cluster. It might not be practical to target 112 genes per cluster because such a low resolution could be less useful for refined analysis. Therefore, we concluded that SOM was superior to MBCluster for this analysis due to the higher correlation coefficient and higher resolution.

### The Robustness of the Integrated Omics Models

The integrated omics models of Pycci 137 and Pycsa 1264 were constructed in order to evaluate the robustness of our method. There were moderate correlations between the transcriptomic and secretomic profiles of the two strains similar to that of Pycco 310 (ρ = 0.55; *p* < 0.001; **Supplementary Table S1**). Similar transcriptomic tendencies were observed among the three strains. There were clusters of nodes highly transcribed only on the complex plant substrates (**Supplementary Figures S1** and **S2**). The genes differentially expressed and co-regulated in response to the complex plant substrates were analyzed using the Pycci 137 and Pycsa 1264 data. A detailed discussion for the selected genes and proteins is provided (**Supplementary Text S1**).

To summarize our findings, Pycci 137 had eight, eleven, and twelve nodes containing genes differentially regulated on wheat straw, pine, and aspen, respectively. As observed with Pycco 310, some genes of Pycci 137 were specifically regulated on pine which could reflect both a metabolic adaptation to this substrate and to the detoxification of the molecules derived from its degradation (e.g., predicted cytochrome P450; protein ID 5741 and 5611).

Meanwhile, Pycsa 1264 had eleven and thirteen nodes that contained genes that were differentially regulated on wheat straw and aspen, respectively. The behavior of Pycsa 1264 was similar to Pycci 137 as the strain showed differential expression of; (1) an AA1_1 laccase (protein ID; 1560767) on the complex plant substrates; and (2) the up-regulation of pectin (GH88; 1428239) and hemicellulose active enzymes including a predicted GH2 mannosidase (1664834) on the complex biomasses (**Supplementary Table S3**; **Supplementary Data S2**)

The three nodes showing the highest numbers of secreted proteins were selected based on the secretomic topography (**Supplementary Figure S2**). The genes coding for the most frequently secreted proteins showed the similar tendencies to Pycco 310 (**Supplementary Table S2**). The growth on Avicel might have triggered the intensive transcription of the genes that led to the production of cellulases and hemicellulases. The selected nodes showed similar repertoires of CAZymes to Pycco 310, which contained a consistent number of CAZymes active on cellulose and glucan polymers associated with a CBM1 module. Notably, CBM1-associated CAZymes were systematically grouped in the same nodes (nodes 14 and 220 for Pycco 310, node 7 for Pycci 137, and node 281 for Pycsa 1264), suggesting that those genes are co-regulated in response to the different substrates.

Overall, similar trends were observed among the three strains. Our method is robust as the biological interpretation of the integrated omics models made sense using the three strains from different fungal species.

## Discussion

### Evaluation of the Integrated Omics Strategies

We demonstrated systematic data mining of genome-wide fungal transcriptomics and secretomics. The purpose was to observe the organisms as a whole, which is different from the conventional gene-by-gene approach.

Simple visual inspection of the integrated omics data enabled us to pinpoint various biological hotspots. Three layers of biological information were presented in our models (**Figures 1**–**3**). The first layer, the mean transcription of biological replicates per substrate, was indicative of the dynamics of the genome-wide transcription responding to the substrates (**Figure 2**). Secondly, the condition-specific transcriptomic patterns showed substrate-wise co-regulation of the genes (**Figure 3C**). Thirdly, the total count of proteins secreted under all conditions facilitated the genome-wide view of which gene clusters actively produced extracellular proteins (**Figure 3B**).

The layers of genome-wide complementary omics information revealed a global picture of the fungal response to the growth conditions. The transcriptomic and secretomic topographies of the three fungal strains were moderately correlated on the genome scale (**Supplementary Table S1**). Thus, our method allowed us to exploit the interconnected omics information by selecting nodes of interest containing co-regulated genes and corresponding gene products.

We demonstrated two different approaches to data mine genes/proteins of interest. The co-regulated genes/proteins were determined by either transcriptomic or secretomic pattern-oriented selection. The former approach unveiled the dynamics of genome-wide transcription patterns in response to the substrates. The latter approach was suitable for identifying enzymes synergistically involved in decomposition of the substrates. Overall, both strategies led to the discoveries of numerous genes of unknown functions and uncharacterized proteins expressed under the specific conditions.

### Comparison of the Clustering Methods

Although MBCluster uses mathematically sophisticated models, the generated gene clusters might be biologically unimportant as the algorithm groups genes based on differential expression patterns (Si et al., 2014). We observed that with this clustering method the genes were clustered according to the similar fold change trends regardless of the scale of the transcription levels (**Supplementary Data S3**). The disregard for high and low transcription levels makes less sense for interpretation of biological phenomena.

Furthermore, we found the design of the method was inflexible in terms of combining omics information and graphic outputs. Branches of the dendrogram can swap and shift making it difficult to grasp the relationship of slightly distant branches. This linear distance-based graphic format could possibly lose some degree of biological relevance.

In contrast, SOMs solved this problem by using six neighbors in proximity, allowing the visual observation of the relationship of the gene clusters on a broad scale. Thus, our models were found to be more biologically significant for the representation of transcriptomics and secretomics, supporting the effectiveness of our approach.

### Construction of Gene Clusters with SOM

It was empirically found that targeting 35 genes in a single node gave optimal resolution of the gene clusters. We observed that when a target of less than 35 genes per node was applied, some nodes had only few or no genes, suggesting a target of 35 genes per node was the lowest limit. The number of genes grouped in one node varied depending on how similar transcriptional patterns were responding to the particular culture conditions. Targeting more than 35 genes per node led to a greater number of genes clustered per node, which decreased the resolution of the SOMs.

We considered genes clustered in a single node as one group. The node-wise selection based on the mean transcription level of all genes within a node was to minimize the loss of biologically relevant information. It treated all genes in a selected node as relevant even if an individual gene in the node had a read count of slightly below 12 log2. Such selection of grouped genes enabled a fuzzy cutoff of around 12 log2 normalized read count for filtering the genes. The idea of grouped genes for comparisons has been successfully demonstrated for microarray analysis (WGCNA; Langfelder and Horvath, 2008).

The default function of a random initialization of weight vectors was applied for simplicity as it does not require additional calculations. Alternatively, linear initialization could be applied with the additional parameters of first eigenvectors corresponding to largest eigenvalues of the input data (Binder and Wirth, 2015). This initialization is suitable for comparisons between separately built SOMs (Steiner et al., 2012). In our case, a single master SOM was constructed for the superimposition of the omics data for comparisons (**Figures 1–3**). The relative arrangement of the SOMs would not be affected regardless of which initialization methods were used.

## Conclusion

We have developed a new method that systematically identifies condition-specific regulated genes and enzymes synergistically involved in the degradation of plant biomass. Our unique strategy was to compress large-scale omics information into simple graphs in order to assist intuitive biological extrapolations. Visual inspection of the topographies made it easy to find molecular hotspots buried in the complex omics data. We have developed the workflow ABCDEFGT to produce a graphic representation of integrated omics data capable of portraying a genome-wide landscape of fungal machineries.

## Materials and Methods

### Fungal Strains, Genomes, RNA Extraction, RNA-Sequencing, Protein Extraction, and Detection

*Pycnoporus coccineus* CIRM-BRFM 310 (herein called Pycco310), *Pycnoporus cinnabarinus* CIRM-BRFM 137 (Pycci 137), and *Pycnoporus sanguineus* CIRM-BRFM 1264 (Pycsa 1264) strains were obtained from the CIRM collection^2^ at the National Institute of Agricultural Research. All three genomes were sequenced by US Department of Energy Joint Genome Institute and annotated using the Joint Genome Institute (JGI) Annotation Pipeline (Grigoriev et al., 2014).

Transcriptome and secretome data were collected from triplicated independent three day-cultures in the presence of either 20 g.l^-1^ maltose, 15 g.l^-1^ Avicel, 15 g.l^-1^ ground wheat straw, 15 g.l^-1^ pine wood, or 15g.l^-1^ aspen wood as the sole carbon source. Three day-cultures were chosen because, by this stage, most of the three day-cultures grown under the all conditions had gone through DNA synthesis, indicating that the fungi were in comparable physiological states (Miyauchi et al., in preparation). The high concentrations of the substrates provided were excess to the growth requirements of the fungal cultures (Miyauchi et al., in preparation). RNA libraries were prepared and sequenced on Illumina HighSeq-2500 (Couturier et al., 2015). The sequence data are available on NCBI (GEO accession: GSE82486). The transcriptome response of Pycsa1264 to pine could not be analyzed due to poor quality of the extracted RNAs from these samples. Secreted proteins were collected from the same cultures, dia-filtered and identified by ESI-MS/MS (Couturier et al., 2015).

### Data Preparation and Manipulations

The obtained Illumina RNA-Seq 150 bp paired end reads were filtered and trimmed using the JGI QC pipeline. QCed reads were aligned to the genomes using TopHat 2 (Kim et al., 2013), counted with HTSeq (Anders et al., 2014), normalized with dsNorm from the DESeq2 Bioconductor package, and log2 transformed (Love et al., 2014). The reliability of the data was verified using a selection of genes by Real Time quantitative PCR (Couturier et al., 2015). Transcriptomic and secretomic data were combined for the data processing. R was used for data manipulations using our customized scripts (R Core Team, 2013).

### SOMs for the Integrated Omics Models

Self-organizing maps were constructed with the R package kohonen (Wehrens and Buydens, 2007). The genes showing similar transcription levels were grouped into nodes of SOMs. It was empirically found that a target of 35 genes per single node of the SOM gave the best resolution of the gene clusters. The default parameters were used for initialization, learning rate, and radius. Hexagonal SOM models were applied to have six neighboring nodes. The map units used were 320, 285, 340 for Pycco 310, Pycci 137, and Pycsa 1264, respectively. The number of iterations used was 100 times more than the map units in order to minimize the Euclidian distances between the nodes for the optimal convergence.

### Genome-Wide Omics Models for the Three Strains

Omics models of the strains were generated by training a SOM with the normalized log2 read count of the fungal responses to maltose, Avicel, pine, aspen, and wheat straw. A topography depicting substrate-specific transcriptomic patterns was comprised of a mean read count of combined biological replicates grown in each carbon source substrate. A topography representing high transcriptional patterns was made by filtering nodes showing a mean read count of the combined triplicates > 12 log2 on each carbon source substrate, which constituted approximately above 75th percentile of the entire gene population. A secretomic topography was created by superimposing the total count of proteins detected mapped onto the SOM nodes.

### Correlation of Transcriptomic and Secretomic Profiles

Spearman’s rank correlation was estimated between the mean transcription level of all conditions (i.e., the node-wise mean transcription topography) and the total count of proteins secreted from all conditions (i.e., the secretomic topography).

### Selection of Genes Showing Substrate-Specific Up-Regulation

Gene clusters (nodes) highly transcribed (>12 log2) on each substrate and commonly highly transcribed (>12 log2) on the substrates were identified. Differentially regulated nodes, which were up-regulated on the complex biomass but not on maltose and Avicel, were determined. Then, the differentially regulated genes were further filtered on the basis of statistically significant log2 fold changes (>1) estimated with DESeq2 (*p* adjusted < 0.05: FDR and Bonferroni correction; Love et al., 2014). The final outputs of the transcriptomics analysis were lists of genes highly transcribed in response to complex plant biomass and differentially regulated as compared to the maltose and Avicel growth conditions.

### Selection of Genes Coding for Frequently Secreted Proteins

The nodes with top three highest counts of proteins were determined from the secretomic topography.

### Comparison of the SOM and MBCluster Methods

The RNA-seq data of Pycco 310 was used for the comparison. Gene clusters were generated with the R package MBCluster.Seq (Si et al., 2014). The number of partitions (*k* = 320) was used to initialize the cluster centroids with the negative binomial model; this number was chosen to match the number of the gene clusters used for SOMs with this fungal strain. The default function of the package, Expectation-Maximization method with negative binomial model was used for clustering. The global mean transcription of the individual clusters was calculated from the results of SOM and MBCluster. The total count of proteins was calculated for the individual clusters. Spearman’s rank correlation coefficients of the transcriptomic and the secretomic profiles were used as an indicator for the biological relevance of the constructed models.

## Author Contributions

SM and M-NR contributed to the manuscript. SM developed the workflow ABCDEFGT. M-NR organized fungal cultures, genome, and RNA sequencing and performed the differential expression analysis. DN, SG, and J-GB performed proteomic analyzes. IG coordinated genome sequencing. RR annotated genomes. AL supervised transcriptome sequencing. DC conducted secretome sequencing. BH performed annotations of CAZymes. All authors have read and approved the manuscript.

## Conflict of Interest Statement

The authors declare that the research was conducted in the absence of any commercial or financial relationships that could be construed as a potential conflict of interest.

## Footnotes

1 www.cazy.org

2 Centre International de Ressources Microbiennes: https://www6.inra.fr/cirm_eng/Filamentous-Fungi

## References

[B1] Amore A. Giacobbe S. Faraco V. (2013). Regulation of cellulase and hemicellulase gene expression in fungi. *Curr. Genomics* 14 230–249. 10.2174/1389202911314040002 24294104 PMC3731814

[B2] Anders S. Pyl P. T. Huber W. (2014). HTSeq – A Python framework to work with high-throughput sequencing data. *Bioinformatics* 31 166–169. 10.1093/bioinformatics/btu638 25260700 PMC4287950

[B3] Binder H. Wirth H. (2015). “Analysis of large-scale OMIC data using self organizing maps,” in *Encyclopedia of Information Science and Technology*, ed. Khosrow-Pour M. (Hershey, PA: Information Resources Management Association), 1642–1653. 10.4018/978-1-4666-5888-2.ch157

[B4] Blanchette R. (1991). Delignification by wood-decay fungi. *Annu. Rev. Phytopathol.* 29 381–398. 10.1146/annurev.py.29.090191.002121

[B5] Couturier M. Navarro D. Chevret D. Henrissat B. Piumi F. Ruiz-Du eñas F. J. (2015). Enhanced degradation of softwood versus hardwood by the white-rot fungus *Pycnoporus coccineus*. *Biotechnol. Biofuels* 8:216 10.1186/s13068-015-0407-8 PMC4683735 26692083

[B6] Couturier M. Navarro D. Olivé C. Chevret D. Haon M. Favel A. (2012). Post-genomic analyses of fungal lignocellulosic biomass degradation reveal the unexpected potential of the plant pathogen *Ustilago maydis*. *BMC Genomics* 13:57 10.1186/1471-2164-13-57 PMC3298532 22300648

[B7] Eggert C. Temp U. Eriksson K.-E. L. (1997). Laccase is essential for lignin degradation by the white-rot fungus *Pycnoporus cinnabarinus.* *FEBS Lett*. 407 89–92. 10.1016/S0014-5793(97)00301-3 9141487

[B8] Floudas D. Binder M. Riley R. Barry K. Blanchette R. A. Henrissat B. (2012). The Paleozoic origin of enzymatic lignin decomposition reconstructed from 31 fungal genomes. *Science* 336 1715–1719. 10.1126/science.1221748 22745431

[B9] Floudas D. Held B. W. Riley R. Nagy L. G. Koehler G. Ransdell A. S. (2015). Evolution of novel wood decay mechanisms in Agaricales revealed by the genome sequences of *Fistulina hepatica* and *Cylindrobasidium torrendii*. *Fungal Genet. Biol.* 76 78–92. 10.1016/j.fgb.2015.02.002 25683379 PMC4399860

[B10] Grigoriev I. V. Nikitin R. Haridas S. Kuo A. Ohm R. Otillar R. (2014). MycoCosm portal: gearing up for 1000 fungal genomes. *Nucleic Acids Res.* 42 D699–D704. 10.1093/nar/gkt1183 24297253 PMC3965089

[B11] Guillén D. Sánchez S. Rodríguez-Sanoja R. (2010). Carbohydrate-binding domains: multiplicity of biological roles. *Appl. Microbiol. Biotechnol.* 85 1241–1249. 10.1007/s00253-009-2331-y 19908036

[B12] Gupta R. Mehta G. Khasa Y. P. Kuhad R. C. (2011). Fungal delignification of lignocellulosic biomass improves the saccharification of cellulosics. *Biodegradation* 22 797–804. 10.1007/s10532-010-9404-6 20711746

[B13] Hernández-Ortega A. Ferreira P. Martnez A. T. (2012). Fungal aryl-alcohol oxidase: a peroxide-producing flavoenzyme involved in lignin degradation. *Appl. Microbiol. Biotechnol.* 93 1395–1410. 10.1007/s00253-011-3836-8 22249717

[B14] Hori C. Gaskell J. Igarashi K. Kersten P. Mozuch M. Samejima M. (2014). Temporal alterations in the secretome of the selective ligninolytic fungus *Ceriporiopsis subvermispora* during growth on aspen wood reveal this organism’s strategy for degrading lignocellulose. *Appl. Environ. Microbiol.* 80 2062–2070. 10.1128/AEM.03652-13 24441164 PMC3993130

[B15] Kersten P. Cullen D. (2014). Copper radical oxidases and related extracellular oxidoreductases of wood-decay Agaricomycetes. *Fungal Genet. Biol.* 72 124–130. 10.1016/j.fgb.2014.05.011 24915038

[B16] Kim D. Pertea G. Trapnell C. Pimentel H. Kelley R. Salzberg S. L. (2013). TopHat2: accurate alignment of transcriptomes in the presence of insertions, deletions and gene fusions. *Genome Biol.* 14:R36 10.1186/gb-2013-14-4-r36 PMC4053844 23618408

[B17] Kohonen T. (1982). Self-organized formation of topologically correct feature maps. *Biol. Cybern.* 43 59–69. 10.1007/BF00337288

[B18] Langfelder P. Horvath S. (2008). WGCNA: an R package for weighted correlation network analysis. *BMC Bioinformatics* 9:559 10.1186/1471-2105-9-559 PMC2631488 19114008

[B19] Langfelder P. Zhang B. Horvath S. (2008). Defining clusters from a hierarchical cluster tree: the Dynamic Tree Cut package for R. *Bioinformatics* 24 719–720. 10.1093/bioinformatics/btm563 18024473

[B20] Levasseur A. Drula E. Lombard V. Coutinho P. M. Henrissat B. (2013). Expansion of the enzymatic repertoire of the CAZy database to integrate auxiliary redox enzymes. *Biotechnol. Biofuels* 6:41 10.1186/1754-6834-6-41 PMC3620520 23514094

[B21] Levasseur A. Lomascolo A. Chabrol O. Ruiz-Dueñas F. J. Boukhris-Uzan E. Piumi F. (2014). The genome of the white-rot fungus *Pycnoporus cinnabarinus*: a basidiomycete model with a versatile arsenal for lignocellulosic biomass breakdown. *BMC Genomics* 15:486 10.1186/1471-2164-15-486 PMC4101180 24942338

[B22] Lloyd S. P. (1982). Least squares quantization in PCM. *IEEE Trans. Inform. Theory* 28 129–137. 10.1109/TIT.1982.1056489

[B23] Lomascolo A. Uzan-Boukhris E. Herpol-Gimbert I. Sigoillot J.-C. Lesage-Meessen L. (2011). Peculiarities of *Pycnoporus* species for applications in biotechnology. *Appl. Microbiol. Biotechnol.* 92 1129–1149. 10.1007/s00253-011-3596-5 22038244

[B24] Lombard V. Golaconda Ramulu H. Drula E. Coutinho P. M. Henrissat B. (2014). The carbohydrate-active enzymes database (CAZy) in 2013. *Nucleic Acids Res.* 42 D490–D495. 10.1093/nar/gkt1178 24270786 PMC3965031

[B25] Love M. I. Huber W. Anders S. (2014). Moderated estimation of fold change and dispersion for RNA-seq data with DESeq2. *Genome Biol.* 15:550 10.1186/PREACCEPT-8897612761307401 PMC4302049 25516281

[B26] MacQueen J. (1967). “Some methods for classification and analysis of multivariate observations,” in *Proceedings of the Fifth Berkeley Symposium on Mathematical Statistics and Probability*, Vol. 1 (Berkeley, CA: Statistics University of California Press), 281–297.

[B27] R Core Team (2013). *R: A Language and Environment for Statistical Computing. R Foundation for Statistical Computing*. Available at: http://www.r-project.org/

[B28] Riley R. Salamov A. A. Brown D. W. Nagy L. G. Floudas D. Held B. W. (2014). Extensive sampling of basidiomycete genomes demonstrates inadequacy of the white-rot/brown-rot paradigm for wood decay fungi. *Proc. Natl. Acad. Sci. U.S.A.* 111 9923–9928. 10.1073/pnas.1400592111 24958869 PMC4103376

[B29] Si Y. Liu P. Li P. Brutnell T. P. (2014). Model-based clustering for RNA-seq data. *Bioinformatics* 30 197–205. 10.1093/bioinformatics/btt632 24191069

[B30] Steiner L. Hopp L. Wirth H. Galle J. Binder H. Prohaska S. J. (2012). A global genome segmentation method for exploration of epigenetic patterns. *PLoS ONE* 7:e46811 10.1371/journal.pone.0046811 PMC3470578 23077526

[B31] Tan P.-N. Steinbach M. Kumar V. (2014). *Introduction to Data Mining.* New York, NY: Pearson Education.

[B32] Wehrens R. Buydens L. M. C. (2007). Self- and Super-organising Maps in R: the kohonen package. *J. Stat. Softw.* 21:29238 10.18637/jss.v021.i05

[B33] Yang I. S. Kim S. (2015). Analysis of whole transcriptome sequencing data: workflow and software. *Genomics Inform.* 13 119–125. 10.5808/GI.2015.13.4.119 26865842 PMC4742321

