# Supplementary material for: Visual Comparative Omics of Fungi for Plant Biomass Deconstruction
Source: Front Microbiol. 2016 Aug 24;7:1335. doi: 10.3389/fmicb.2016.01335 (PMC4996036; doi:10.3389/fmicb.2016.01335)
Supplement: Supplementary file 2 [file Image_1.PDF]

Supplementary Material

Visual comparative omics of fungi for plant biomass deconstruction

Shingo Miyauchi*, David Navarro, Igor V. Grigoriev, Anna Lipzen, Robert Riley, Didier Chevret, Sacha Grisel, Jean-Guy Berrin, Bernard Henrissat, Marie-Noëlle Rosso

*** Correspondence:** Shingo Miyauchi, Marie-Noëlle Rosso: shingo.miyauchi@univ-amu.fr, marie-noelle.rosso@univ-amu.fr

1 Supplementary Data

#	*Files*	*Descriptions*
Data S1	Pycco310_Transcriptome&Secretome_per_node.xlsx<br>Pycci137_Transcriptome&Secretome_per_node.xlsx<br>Pycsa1264_Transcriptome&Secretome_per_node.xlsx	The output files used for the estimation of Spearman's rank correlation between transcriptome and secretome of the three *Pycnoporus* strains.
Data S2	supplementaryTable_selectedGenes_pycco.aspen.xlsx<br>supplementaryTable_selectedGenes_pycco.pine.xlsx<br>supplementaryTable_selectedGenes_pycco.wheatstraw.xlsx<br>supplementaryTable_selectedGenes_pycci.aspen.xlsx<br>supplementaryTable_selectedGenes_pycci.pine.xlsx<br>supplementaryTable_selectedGenes_pycci.wheatstraw.xlsx<br>supplementaryTable_selectedGenes_pycsa.aspen.xlsx<br>supplementaryTable_selectedGenes_pycsa.wheatstraw.xlsx	The lists of the genes selected for the potential involvement in the decomposition of plant substrates for the three *Pycnoporus* strains.
Data S3	Pycco310_MBCluster_list_100.xlsx<br>Pycco310_MBCluster_list_320.xlsx<br>Pycco310_MBCluster_Transcriptome&Secretome_100.xlsx<br>Pycco310_MBCluster_Transcriptome&Secretome_320.xlsx	1) Results of 320 and 100 clusters generated with MBCluster.seq. The lists of genes with protein ID, log2 transformed read counts, log2 fold changes from the differential expression analysis.<br>2) The output files for the calculation of Spearman's rank correlation between transcriptome and secretome for 320 and 100 clusters above.

2 Supplementary Figures

Figure S1: The integrated omics models of Pycci 137 and Pycsa 1264. **Pycci 137**: *Pycnoporus cinnabarinus CIRM-BRFM 137*. **Pycsa 1264**: *Pycnoporus sanguineus CIRM-BRFM 1264*. The node identification is labeled (i.e. 1 to 270). **a**: Mean transcription of biological replicates for the individual substrates. **b**: Clusters of highly transcribed genes (>12 log2 read count) were highlighted in circle. Grey: Common to all substrates. Light green: Maltose. Purple: Avicel. Pink: Apen, wheat straw, pine. **NOTE**: No RNA-seq data available for Pycsa1264 grown on pine.

Pycci137

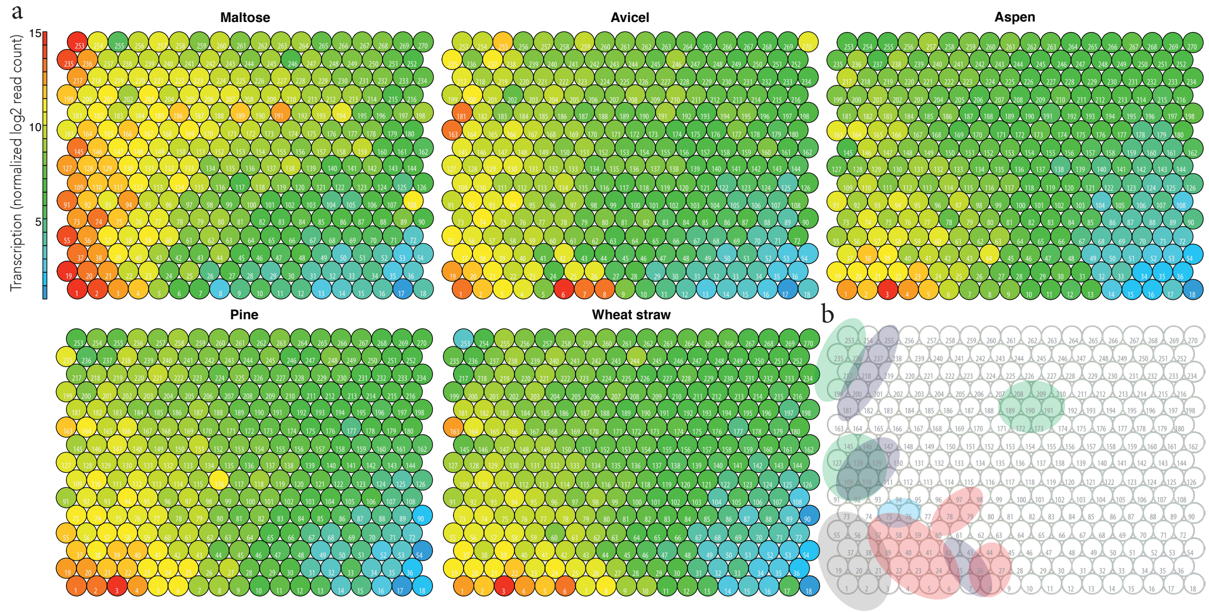

Pycsa1264

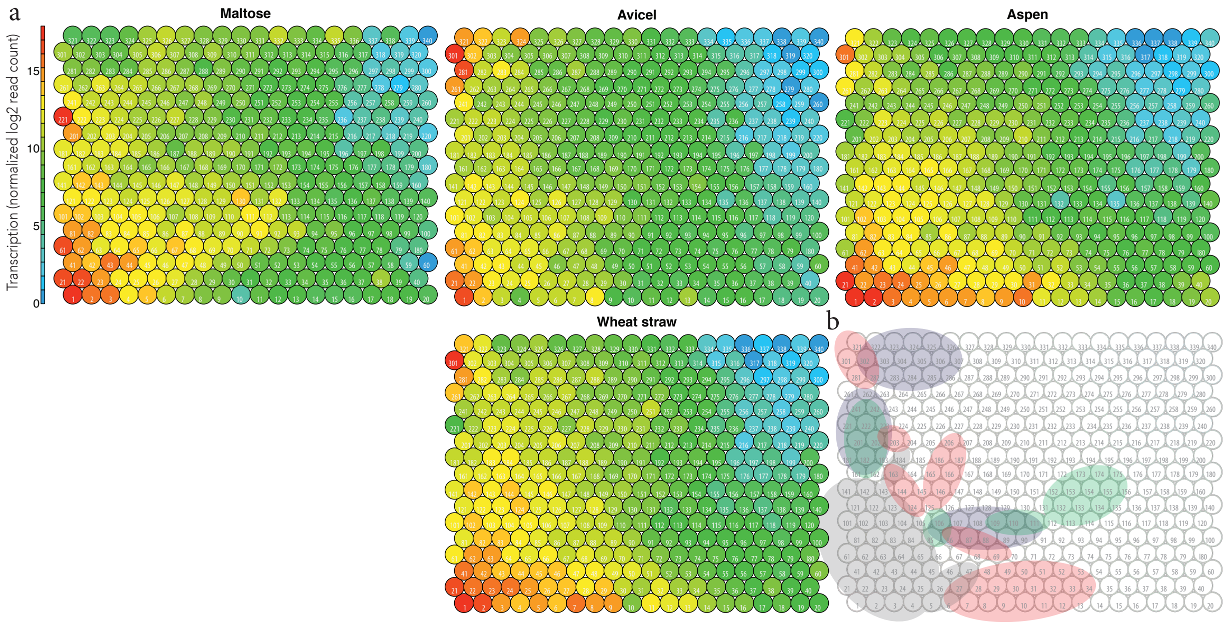

Figure S2: The integrated omics models of Pycci 137 and Pycsa 1264. **Pycci 137**: *Pycnoporus cinnabarinus* CIRM-BRFM 137. **Pycsa 1264**: *Pycnoporus sanguineus* CIRM-BRFM 1264. **a**: The genome-wide mean transcription of the genes from all conditions. **b**: The count of the total proteins secreted showing hotspots. **c**: The groups of substrate-specific highly transcribed genes (>12 log2 read count) were labeled and highlighted. **C**: Common to all substrates. **M**: Maltose. **Av**: Avicel. **A**: Aspen. **P**: Pine. **W**: Wheat straw. **NOTE**: No RNA-seq data available for Pycsa 1264 grown on pine.

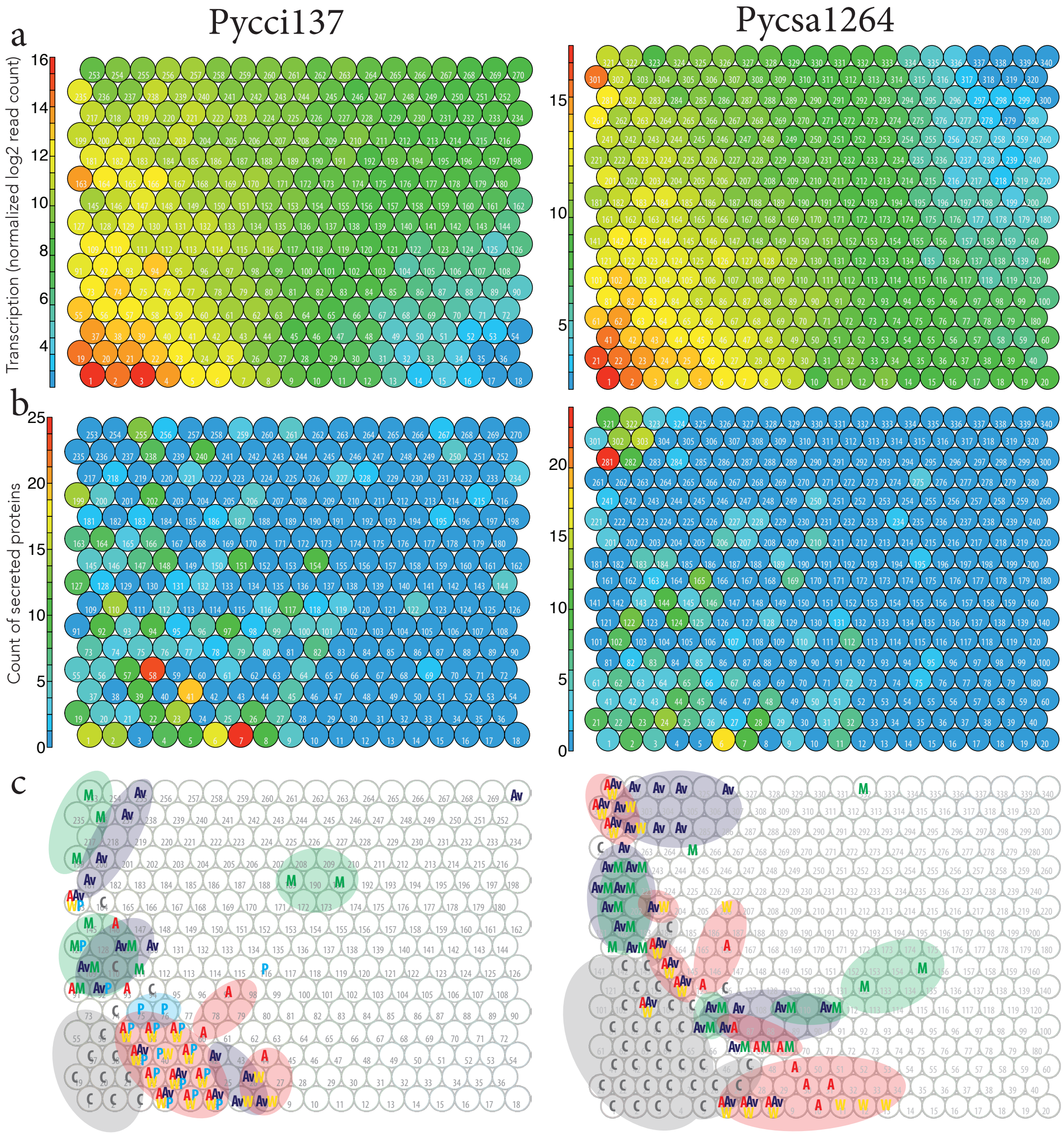

3 Supplementary Tables

Table S1: Spearman's rank correlation of genome-wide transcriptome and secretome for the three *Pycnoporus* strains. Spearman's rank correlation coefficients were calculated from the node-wise mean transcription and the total count of secreted proteins for the three *Pycnoporus* strains. The coefficients for the three strains were approximately 0.55 ($p < 0.001$). The output files used for the calculation are available (Data S1).

Strain	*Correlation coefficient*
Pycco 310	0.55
Pycci 137	0.56
Pycsa 1264	0.55

Table S2: Selection of nodes containing genes coding for frequently secreted proteins. **ProteinID**: The ID for the proteins predicted by JGI. **NodeID**: The ID of the nodes shown in the genome-wide transcriptomic and secretomic integrated models. **Proteins secreted:** the presence of proteins detected from the 3rd day of fungal cultures using LC-MS. **ASP**: Aspen. **PIN**: Pine. **WHS**: Wheat straw. **AVI:** Avicel. **MAL:** Maltose. **Mal/Avi/Asp/Pin/Whs.ave**: The mean of the normalized log2 read count combined from the biological replicates cultivated on maltose/Avicel/aspen/pine/wheat straw. **Annotations:** The information including CAZymes and KOG descriptions obtained from the prediction of the annotations by JGI. **Uncharacterized proteins**: Lines highlighted in pink. **NOTE**: No RNA-seq data available for Pycsa 1264 grown on pine.

Pycnoporus coccineus BRFM 310 (Pycco 310)

ProteinID	nodeID	Proteins secreted					Mal.ave	Avi.ave	Asp.ave	Pin.ave	Whs.ave	Annotations
1369367	120	ASP	PIN	WHS	AVI	MAL	11	15	14	12	14	GH47
1373467	120	ASP	PIN	WHS	AVI	MAL	12	15	14	13	13	GH92
1431843	120	ASP	PIN	WHS	AVI	MAL	12	14	13	14	13	GH27
1433845	120	ASP	PIN	WHS	AVI	MAL	12	15	14	13	12	GH17
1438510	120					MAL	12	14	13	13	13	-
1439714	120						12	15	14	13	15	20S proteasome, regulatory subunit beta type PSMB1/PRE7
1460251	120						11	15	13	12	13	GH5_22
1466845	120	ASP	PIN	WHS	AVI	MAL	12	15	13	13	13	PL8_4
1470072	120						13	15	13	12	12	-
1357326	14	ASP	PIN	WHS	AVI		7	16	15	14	12	CBM1-GH6
1359888	14	ASP	PIN	WHS	AVI		8	15	14	14	13	CBM1-GH5_7
1382161	14						8	16	14	12	12	AA9-CBM1
1389216	14	ASP	PIN	WHS	AVI	MAL	7	16	13	12	12	GH7
1401955	14	ASP	PIN	WHS	AVI		8	16	14	13	14	AA8-AA3_1
1426831	14						7	14	14	12	12	GH115
1428145	14			WHS			9	16	14	12	13	AA9-CBM1
1439328	14			WHS	AVI		6	14	13	14	14	CBM1-CE16
1467772	14	ASP	PIN	WHS	AVI		7	16	13	12	12	GH131-CBM1
378107	35	ASP	PIN	WHS	AVI	MAL	7	12	12	12	12	-
1370654	35	ASP	PIN	WHS	AVI		8	12	13	13	13	GH28
1370768	35		PIN				8	12	13	14	13	Lysophospholipase
1372078	35						7	12	13	11	12	-
1378088	35						7	13	13	12	13	Cystathionine beta-lyases/cystathionine gamma-synthases
1405174	35						8	12	13	12	13	-
1411666	35	ASP	PIN	WHS	AVI		8	13	12	12	12	GH3
1424462	35						6	12	13	13	13	RNA polymerase II C-terminal domain-binding protein RA4, contains RPR and RRM domains
1429888	35						6	11	13	12	12	AA3_2
1433077	35	ASP	PIN	WHS			5	13	13	13	12	GH45
1434242	35						7	11	13	13	12	-
1437875	35				AVI		7	12	13	14	13	Serine-type carboxypeptidases
1444114	35	ASP	PIN	WHS	AVI	MAL	6	13	14	12	15	Tripeptidyl-peptidase I
1474390	35						6	12	13	11	13	
1366028	220	ASP	PIN	WHS	AVI		7	15	13	9	11	GH7
1373375	220	ASP	PIN		AVI	MAL	8	13	12	11	10	CE16
1375723	220			WHS	AVI		6	14	12	11	8	GH12
1392142	220	ASP		WHS	AVI		8	12	12	9	9	CBM1-CE1
1404940	220	ASP	PIN	WHS	AVI		9	13	11	9	9	CBM1-GH3
1434718	220	ASP	PIN	WHS			6	14	13	9	9	CBM1-GH5_5
1468081	220						7	13	11	10	9	cerato-plantanin
1470260	220			WHS	AVI		7	13	11	10	10	CBM1-CE15
1472584	220			WHS			7	14	12	10	9	CBM1

Pycnoporus cinnabarinus BRFM 137 (Pycci 137)

proteinID	*nodeID*	*Proteins secreted*					*Mal.ave*	*Avi.ave*	*Asp.ave*	*Pin.ave*	*Whs.ave*	*Annotations*
1365	7	ASP		WHS	AVI		6	16	10	10	13	GH131-CBM1
1929	7	ASP		WHS	AVI		6	15	10	10	13	CBM1-GH5_5
3419	7	ASP		WHS	AVI		7	15	10	10	13	Predicted mutarotase
3419	7	ASP		WHS	AVI		7	15	10	10	13	Predicted mutarotase
4378	7						7	16	11	11	13	GH1
5579	7			WHS			7	15	10	9	12	AA9-CBM1
5845	7	ASP		WHS	AVI		8	17	9	9	12	GH7
6394	7						7	15	12	11	13	-
6605	7	ASP	PIN	WHS	AVI	MAL	7	13	11	10	14	Carboxypeptidase C
8616	7	ASP	PIN	WHS	AVI		8	17	10	10	12	CBM1-GH5_5
65	58						10	12	13	12	12	-
598	58	ASP	PIN	WHS	AVI	MAL	10	11	13	12	13	GH92
929	58	ASP	PIN	WHS	AVI	MAL	10	11	13	14	12	GH95
1192	58						10	10	14	12	12	Cytochrome P450 CYP4/CYP19/CYP26 subfamilies
1284	58						10	10	12	12	12	Alkyl hydroperoxide reductase, thiol specific antioxidant and related enzymes
1924	58						10	10	13	12	12	Inorganic phosphate transporter
3446	58	ASP	PIN	WHS	AVI	MAL	10	10	12	11	14	AA1_1
3585	58						10	11	12	13	12	Uncharacterized protein PSP1 (suppressor of DNA polymerase alpha mutations in yeast)
3798	58	ASP		WHS			10	10	13	12	13	GH28
4353	58						10	11	13	13	12	Multidrug/pheromone exporter, ABC superfamily
4662	58						10	10	13	13	12	-
6135	58						11	10	13	13	12	-
6333	58						11	11	13	12	12	-
7058	58						10	11	13	13	12	Aquaporin (major intrinsic protein family)
7210	58						10	11	13	12	12	-
7290	58						10	10	13	12	13	Amidases
7533	58						10	10	12	13	12	Kynurenine 3-monooxygenase and related flavoprotein monooxygenases
7593	58	ASP	PIN	WHS	AVI	MAL	10	11	14	13	13	-
8051	58						10	11	14	13	11	Cystathionine beta-lyases/cystathionine gamma-synthases
8195	58						11	11	13	13	12	Chitinase
8220	58			WHS			10	12	13	13	13	GH88
8493	58						10	12	13	12	13	Cytochrome P450 CYP4/CYP19/CYP26 subfamilies
1092	41	ASP	PIN	WHS	AVI	MAL	8	9	12	12	12	GH2
3335	41						10	8	12	13	13	Aldo/keto reductase family proteins
4566	41	ASP	PIN	WHS		MAL	9	9	11	13	13	GH3
5077	41	ASP	PIN	WHS	AVI	MAL	9	8	12	13	12	-
6441	41						8	9	13	13	13	-
7930	41						10	9	13	14	12	Aquaporin (major intrinsic protein family)
9738	41	ASP	PIN	WHS	AVI	MAL	10	9	12	12	13	GH31

Pycnoporus sanguineus BRFM 1264 (Pycsa 1264)

proteinID	*nodeID*	*Proteins secreted*					*Mal.ave*	*Avi.ave*	*Asp.ave*	*Whs.ave*	*Annotations*
571793	281	ASP		WHS	AVI		9	17	12	13	GH131-CBM1
1567787	281	ASP	PIN	WHS	AVI		8	18	14	15	CBM1-GH6
1583489	281			WHS	AVI		9	18	13	14	AA9-CBM1
1672345	281	ASP	PIN	WHS	AVI		9	16	13	14	CBM1-GH5_7
1672751	281	ASP	PIN	WHS	AVI		9	17	13	15	AA8-AA3_1
1675671	281	ASP	PIN	WHS	AVI	MAL	10	20	11	13	GH7
1721886	281	ASP	PIN	WHS	AVI		10	17	13	14	GH74
1738933	281	ASP	PIN	WHS	AVI		10	17	13	14	GH28
1608570	6	ASP	PIN	WHS	AVI	MAL	11	12	14	14	Serine carboxypeptidases (lysosomal cathepsin A)
1609201	6	ASP		WHS	AVI		12	13	14	14	GH47
1615305	6						12	13	13	13	-
1641584	6						11	13	15	14	UDP-glucuronosyl and UDP-glucosyl transferase
1645517	6			WHS			12	13	14	13	GH17
1660524	6						12	12	13	14	Predicted GTPase-activating protein
1663870	6	ASP	PIN	WHS	AVI	MAL	10	12	14	14	GH92
1674723	6						11	12	14	14	-
1678028	6						12	13	13	14	Predicted alpha/beta hydrolase
1682233	6						11	13	14	14	GH13_1
1758816	6						11	12	15	13	-
1183761	303	ASP		WHS	AVI		9	12	11	11	-
1210095	303						7	12	10	11	-
1561085	303	ASP		WHS			8	11	11	12	GH43
1561531	303						8	11	11	11	-
1561925	303	ASP	PIN	WHS		MAL	9	11	11	12	GH79
1566927	303						9	12	11	11	-
1577052	303			WHS			8	11	11	11	GH3
1577721	303	ASP	PIN	WHS	AVI		7	13	10	12	CE8
1601452	303	ASP			AVI	MAL	8	12	10	12	GH18
1668510	303						8	11	11	11	UDP-glucuronosyl and UDP-glucosyl transferase
1681942	303						9	12	11	12	-

Table S3: The summary of the selected co-regulated genes of Pycci 137 and Pycsa 1264 on aspen, pine, and wheat straw. **Biomass-specific nodes/genes**: The number of nodes containing genes with higher transcription on aspen, pine, and wheat straw than maltose and cellulose. High transcription was defined as >12 log2 average read counts of the node on each biomass. **Unique nodes/genes:** The number of nodes containing genes highly transcribed on a single complex biomass (either aspen, pine, or wheat straw). **Genes selected**: The number of highly transcribed genes was determined by the further filtration based on the differential gene expression (log2 fold changes >1) with statistical significance (adjusted $p < 0.05$). **Secretion detected**: The secretion of proteins detected. The ratio of the detected proteins divided by the total genes is shown in the brackets. **CAZyme**: Carbohydrate-active enzymes. **Unknown genes**: genes with no functional annotations. **Unknown secreted**: Uncharacterized proteins identified in the secretomes. The detailed lists are available (Data S2). **NOTE**: No RNA-seq data available for Pycsa 1264 grown on pine.

Pycnoporus cinnabarinus BRFM 137 (Pycci 137)

Biomass	*Biomass-specific nodes / genes*	*Unique nodes / genes*	*Genes selected*	*Secretion detected*	*CAZymes*	*Unknown genes*	*Unknown secreted*
Aspen	12 / 159	5 / 37	117	27 (23.0%)	AA1_1, EXPN, GH2, GH3, GH5_9, GH13_1, GH18, GH18CBM5, GH27, GH28, GH31, GH35, GH79, GH88, GH92, GH95, GH125	31	2
Pine	11 / 194	3 / 65	124	24 (17.9%)	AA1_1, AA3_2, EXPN, GH2, GH3, GH5_9, GH13_1, GH18, GH27, GH28, GH31, GH35, GH37, GH88, GH92, GH95, GH92, GT4, GT32	31	2
Wheat straw	8 / 129	-	78	21 (26.9%)	AA1_1, EXPN, GH2, GH3, GH5_9, GH13_1, GH18, GH27, GH28, GH31, GH35, GH88, GH92, GH95, GH125	19	1

Pycnoporus sanguineus BRFM 1264 (Pycsa 1264)

Biomass	*Biomass-specific nodes / genes*	*Unique nodes / genes*	*Genes selected*	*Secretion detected*	*CAZymes*	*Unknown genes*	*Unknown secreted*
Aspen	13 / 229	6 / 91	91	15 (16.5%)	AA1_1, CE16, GH16, GH18, GH18-CBM5, GH43, GH88, GH92, GH125	30	1
Wheat straw	11 / 179	4 / 41	88	20 (22.7%)	CE15, GH2, GH3, GH18, GH18-CBM5, GH28, GH43, GH76, GH88, GH92, GH95, GH125, PL8_4	21	1

4 Supplementary Texts

Text S1: Detailed descriptions of the profiles of Pycci 137 and Pycsa 1264 for the selection of genes regulated in substrate-specific manners and the selection of nodes enriched in secreted proteins.

We focused on the genes from Pycci 137 and Pycsa 1264 that were differentially expressed and co-regulated in response to the plant substrates (i.e. aspen, pine, and wheat straw) in contrast to the simple substrates (i.e. maltose and cellulose; data S2; table S3).

In the case of Pycci 137, we identified eight, eleven and twelve nodes contained genes differentially regulated on wheat straw, pine, and aspen, respectively. Of these, seven nodes were commonly found in response to the three substrates. Those nodes were enriched in genes coding for CAZymes potentially active on the plant biomass. While the nodes selected from Pycco 310 contained several genes coding for family AA2 lignin-active enzymes, the nodes selected from Pycci 137 did not contain AA2 but did contain two genes coding for AA1_1 laccases (protein ID 8672; 3446). Laccases are thought to contribute to lignin depolymerization through the oxidation of lignin-derived phenolics (Munk et al., 2015). Consistent with the absence of AA2 peroxidases in the selected nodes, no gene coding for AA3_2 or AA5_1 auxiliary oxidoreductases was selected.

The selected nodes contained genes coding for pectin-active enzymes GH28 (protein ID 8001; 3798), GH88 (8220), and hemicellulose such as a GH27 predicted arabinosidase (7245) and a GH95 predicted xyloglucanase (929). A gene coding for a predicted GH2 mannosidase (1092) was found in the nodes regulated on the three substrates despite only pine wood containing this saccharide, suggesting that this gene is not specifically regulated in response to mannan, but is rather co-regulated with other biomass degrading enzymes. Among the co-regulated genes, some of them were predicted as cytochrome P450 genes, suggesting common mechanisms for metabolization of the degradation products by the fungus (protein IDs 2161; 8493; 1192). Some proteins that were predicted for secretion were notably co-regulated with biomass degrading CAZymes (e.g. protein IDs 1391; 2712; 4662). As observed for Pycco 310, some genes were specifically regulated on pine that could reflect a metabolic adaptation to this substrate, and to the detoxification of the molecules derived from its degradation (e.g. predicted cytochrome P450; protein ID 5741 and 5611).

In the case of Pycsa 1264, we identified 11 and 13 nodes that contained genes differentially regulated on wheat straw and aspen respectively. The behavior of Pycsa 1264 was similar to Pycci 137 as Pycsa 1264 showed differential expression of an AA1_1 laccase (protein ID 1560767) on the plant substrates and the up-regulation of pectin (GH88; 1428239) and hemicellulose active enzymes including a predicted GH2 mannosidase (1664834) on the plant substrates.

The three nodes containing the highest number of proteins detected in the secretome were selected from the secretomic topography of Pycci 137 and Pycsa 1264 (Table S2). The genes coding for the most frequently secreted proteins showed the similar tendencies to Pycco 310. The presence of Avicel might have triggered the intensive transcription of the genes that led to the production of cellulases and hemicellulases for all the strains.

The selected nodes showed similar CAZyme contents as Pycco310, and contained a consistent number of CAZymes associated with a CBM1 module active on cellulose and glucan polymers. Notably, the genes coding for CBM1-associated CAZymes were systematically grouped in the same

nodes (nodes 14 and 220 for Pycco 310, node 7 for Pycci 137, node 281 for Pycsa 1264), suggesting that those genes are co-regulated in response to the different substrates. As observed from the transcriptome analyses, we also detected AA1_1 predicted laccases and GH2 predicted mannosidase in the secretomes of Pycci 137. Furthermore, an AA8-AA3 predicted protein was found in the secretomes of the three strains and the corresponding genes belong to nodes that were enriched in secreted proteins. AA8-AA3 CAZymes, which are modular enzymes, correspond to cellobiose dehydrogenases. They act in synergy with AA9 Lytic Polysaccharide Monooxygenases for the oxidative cleavage of glycosidic bonds (Bennati-Granier et al., 2015). The association of AA8-AA3 and AA9 genes in a same node in Pycco 310 (node 14) and in Pycsa 1264 (node 281) shows that the genes are co-regulated therefore strengthening the hypothesis of synergetic activities *in vivo*.

References

1. Bennati-Granier, C., Garajova, S., Champion, C., Grisel, S., Haon, M., Zhou, S., et al. (2015). Substrate specificity and regioselectivity of fungal AA9 lytic polysaccharide monooxygenases secreted by *Podospora anserina*. *Biotechnol. Biofuels* 8, 90. doi:10.1186/s13068-015-0274-3.
2. Munk, L., Sitarz, A. K., Kalyani, D. C., Mikkelsen, J. D., and Meyer, A. S. Can laccases catalyze bond cleavage in lignin? *Biotechnol. Adv.* 33, 13–24. doi:10.1016/j.biotechadv.2014.12.008.
